# Development of a Fluorescence Polarization Based High-Throughput Assay to Identify Casitas B-Lineage Lymphoma RING Domain Regulators

**DOI:** 10.1371/journal.pone.0078042

**Published:** 2013-10-31

**Authors:** Xingliang Xie, Lin Sun, Ziyan Yuan Pessetto, Yan Zhao, Zhihe Zang, Ling Zhong, Min Wu, Qing Su, Xiurong Gao, Wang Zan, Yiyi Sun

**Affiliations:** 1 Department of Pharmacy, Chengdu Medical College, Chengdu, Sichuan Province, China; 2 West China Hospital, Sichuan University, Chengdu, Sichuan Province, China; 3 Department of Pathology & Laboratory Medicine, Univerisity of Kansas Medical Center, Kansas City, Kansasa, United States of America; Russian Academy of Sciences, Institute for Biological Instrumentation, Russian Federation

## Abstract

The E3 ubiquitin protein ligase Casitas B-lineage Lymphoma (Cbl) proteins and their binding partners play an important role in regulating signal transduction pathways. It is important to utilize regulators to study the protein-protein interactions (PPIs) between these proteins. However, finding specific small-molecule regulators of PPIs remains a significant challenge due to the fact that the interfaces involved in PPIs are not well suited for effective small molecule binding. We report the development of a competitive, homogeneous, high-throughput fluorescence polarization (FP) assay to identify small molecule regulators of Cbl (RING) domain. The FP assay was used to measure binding affinities and inhibition constants of UbCH7 peptides and small molecule regulators of Cbl (RING) domains, respectively. In order to rule out promiscuous, aggregation-based inhibition, two assay conditions were developed and compared side by side. Under optimized conditions, we screened a 10,000 natural compound library in detergent-free and detergent-present (0.01% Triton X-100) systems. The results indicate that the detergent-present system is more suitable for high-throughput screens. Three potential compounds, methylprotodioscin, leonuride and catalpol, have been identified that bind to Cbl (RING) domain and interfere with the Cbl (RING)-UbCH7 protein-protein interaction.

## Introduction

The casitas B-lineage Lymphoma (Cbl) proteins have created much interest in recent years. So far seven members of Cbl-family proteins have been characterized including three mammalian isoforms (c-Cbl, Cbl-b, Cbl-c/Cbl-3), two isoforms in *Drosophila melanogaster* (Fly Cbl, short and long), *Caenorhabditis elegans* (SLI-1), and chick Cbl [1], [2]. In addition, there are four known oncogenic Cbl proteins including v-Cbl, 70Z-Cbl, ΔY368-Cbl and ΔY371-Cbl [1] (**Fig. S1**). The oncogenic protein v-Cbl, the first member of the Cbl-family proteins, was cloned from the Cas NS-1 retrovirus in 1989 [3]. Later, it was subsequently discovered that v-Cbl is a truncated form of a larger cellular homologue known as c-Cbl [4]. c-Cbl is a ubiquitously expressed, primarily cytosolic protein which has 906 and 913 amino acids in humans and mice, respectively [5]. The full length of c-Cbl consists of an N-terminal tyrosine-kinase-binding (TKB) domain, a RING Finger motif, a proline-rich region and a C-terminal ubiquitin-associated (UBA) domain that overlaps with a LEUCINE ZIPPER (LZ) motif [1].

By virtue of its E3 ubiquitin ligase activity, c-Cbl is widely known as a negative regulator of growth factor action. Signaling proteins known to be targeted for ubiquitination by c-Cbl proteins include EGF receptor, PDGF receptor, Macrophage Colony-Stimulating Factor (M-CSF) receptor, Neu receptor, Epidermal Growth Factor-2 (ErbB-2) receptor, c-Src, c-Cbl, and T-cell and B-cell antigen receptor [6], [7]. Inhibition of this process results in enhanced signal transduction [1]. In addition to the E3 ubiquitin ligase activity, c-Cbl has been reported to act as an adaptor protein in a range of intracellular signaling cascades [1], [8].

The Cbl family of ubiquitin ligases, along with a set of phosphotyrosine-binding adaptors, integrates receptor endocytosis into the densely wired networks of signal transduction, which plays a vital role in health and disease. However, the mechanism is not well understood due to limited techniques to dissect Cbl protein functions. Here we designed a competitive, homogeneous, high-throughput FP assay to identify small molecule regulators that binds to Cbl (RING) domain.

## Materials and Methods

### Peptides and Proteins

Peptides (**Table 1**) were purchased from Pharmanic (Chengu, Sihucna, China) with a purity ≥98%. The peptide concentrations were determined by amino acid analysis at the protein core facility (Chengdu Medical College). The recombinant Cbl protein (**Fig. S2**) was produced by the protein core at Chengdu Medical College by the PCR technique as described by Yokouchi *et al* [9]. c-Cbl (RING) protein including the linker and RING domain (codon 358–457) was tagged with glutathione S-transferase (GST) and purified with GSH-Sepharose (Shanghai Biopharma).

**Table 1:** Probes used in FP assay.

Peptide	Sequence
Probe **1**	FITC-βA-P**F**KPP-NH_2_
Probe **2**	FITC-βA-K**P**ATK-NH_2_
Peptide **1**	Ac-P**F**KPP-NH_2_
Peptide **2**	Ac-P**A**KPP-NH_2_

### Compound Library

The natural product library contains 10,000 natural products with a minimum of 98% purity confirmed by NMR and HPLC (Pharmanic, Chengdu, Sichuan, China). Compounds are selected according to Pharmacopoeia of the People’s Republic of China. All compounds are approved by China Food and Drug Administration as Chinese Traditional Medicine. Compounds were present at 10 mmol/L in DMSO. Compounds were seeded from A2 to H11 in 96-well plates.

### Reagents

All the chemicals are purchased from Kepeng Ltd (Beijing, China) except specific indication.

### FP Assay Development

Assays were carried out in detergent-free buffer system (PBS, pH = 7.4) or detergent-present buffer system (0.01% Triton X-100 in PBS, pH = 7.4). All FP measurements were made on 384-well, low-volume, black, v-bottom polystyrene microplates (Corning, Shanghai, China) using the ZS-2 plate reader. The binding affinities of fluorescein-labeled peptides/regulators binding to c-Cbl (RING) protein were verified by titrating an increasing concentration of protein into a constant concentration of labeled peptides. The polarization values were measured at an excitation wavelength of 485 nm and an emission wavelength of 538 nm. The total fluorescence (TF) values were also measured. The change in polarization was graphed as a function of the log of the protein concentration, and the dissociation constant (K_d_) and the change in mP (ΔmP) were obtained from the resulting sigmoidal curve. The concentrations of fluorescein-labeled peptide and proteins were optimized.

### FP Assay Stability Assessment

Unlabeled short peptide was titrated against the FITC-labeled peptide (100 nmol/L) and protein (3 µmol/L) mixture. Plates were incubated at room temperature for up to 3 hours or 30 min at different temperatures. FP values were taken and IC_50_ values, concentrations giving 50% inhibition of protein-protein interaction with respect to control, and dissociation constant (K_d_) values were calculated.

### DMSO Tolerance Assay

Increasing concentrations of DMSO at 1–20% of the assay volume (20 µl) were added to the FITC-labeled peptide and protein mixture. FP and TF measurements were taken at room temperature (25°C) after 30 min incubation.

### FP Based Screen

Master mixtures containing fluorescein-labeled probe and Cbl (RING) protein Cbl (RING) protein, 3 µmol/L and probe **1** at 100 nmol/L) in a reaction volume of 19 µl were prepared and loaded to plates. The library compounds (10 µmol/L – 0.078 µmol/L, half dilutions) were prepared separately and transferred into the assay plates. The first and last columns of each plate were used as positive and negative control wells. Wells containing the drug only were used as controls to detect auto-fluorescence of the compounds in the total fluorescence assay. After subtraction of the background signal, data was processed and the IC_50_ values were determined by non-linear least square fitting (four parameters) using SigmaPlot 11.0.

### Hits Validation

Hits were validated using the primary screening assay conditions in triplicate.

### Statistical Analysis

Data were reported as mean ± SD. Each experiment was performed 3–5 times, each treatment performed in duplicate or triplicate.

## Results

### Screen Assay Development

We hypothesized that unlabeled or fluorescein-labeled peptides (Peptide **1**, Probe **1** and **2**, **Table 1**) derived from UbCH7 structure bind to the Cbl (RING) domain and can be used to identify compounds/chemical regulators that competitively bind to the Cbl (RING) domain.

**Figure 1:**
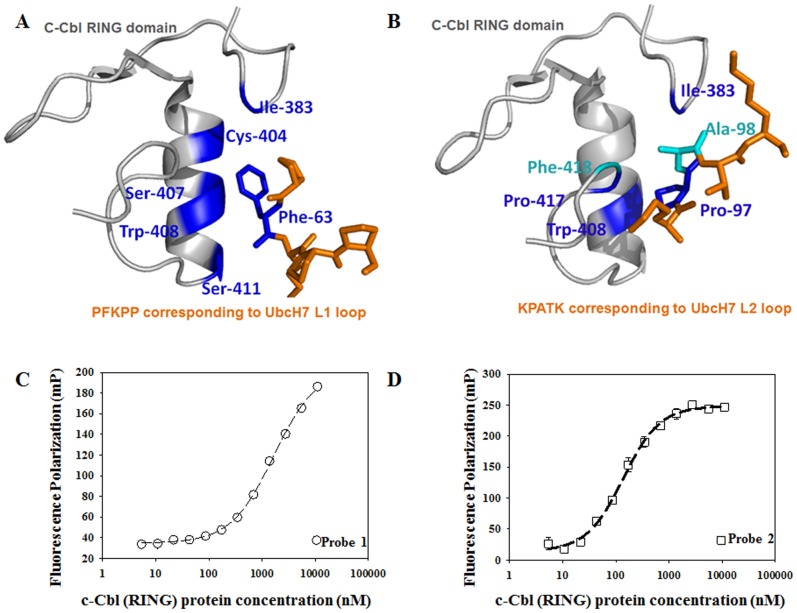
Assay development. A. The structure of PFKPP derived from UbCH7 L1 loop and c-Cbl RING domain complex. B. The structure of KPATK derived from UbcH7 L2 loop and c-Cbl RING domain complex (PDB ID: 1FBV). C. c-Cbl (RING) – Probe 1 peptide binding isotherms: an increasing concentration of protein (5 nmol/L to 11 µmol/L) was titrated into 100 nmol/L probe probe 1. K_d_ = 1.67±0.09 µmol/L; Δmp = 150 units. D. c-Cbl (RING) – Probe 2 peptide binding isotherms: an increasing concentration of protein (5 nmol/L to 11 µmol/L) was titrated into 100 nmol/L probe 2. K_d_ = 0.14±0.08 µmol/L; Δmp = 230 units.

The FP assay utilizing fluorescein-labeled peptides binding to the Cbl (RING) domain was developed and optimized in a 384-well format. Two probes were designed to determine the effect of FITC on the assay. Binding experiments were performed and K_d_ values were calculated. Titration of c-Cbl (RING) protein with these probes causes the FP and the fluorescence anisotropy (FA) to increase 4 to 6-fold. Binding affinities to probe **1** or probe **2** were evaluated (**Fig. 1C&D**). Probe **2** binds to c-Cbl (RING) protein with a higher affinity (K_d_ = 0.14±0.08 µmol/L, **Fig. 1D**) than probe **1** (K_d_ = 1.67±0.09 µmol/L, **Fig. 1C**). To study competitive inhibition using the FP binding assay, the concentration ratio between the receptors and the K_d_ of the labeled probes should be at least 1 in order to generate IC_50_ curve [10]. Therefore, probe **1** was chosen for the following validation and screen assays. In addition, the receptor concentration needed for the FP assay should be ≥ K_d_ and should have a reasonable ΔmP. The ΔmP value was about 100 units when 3 µmol/L c-Cbl (RING) protein was titrated. Therefore, 3 µmol/L was used as the c-Cbl (RING) protein concentration for the following screen.

**Figure 2:**
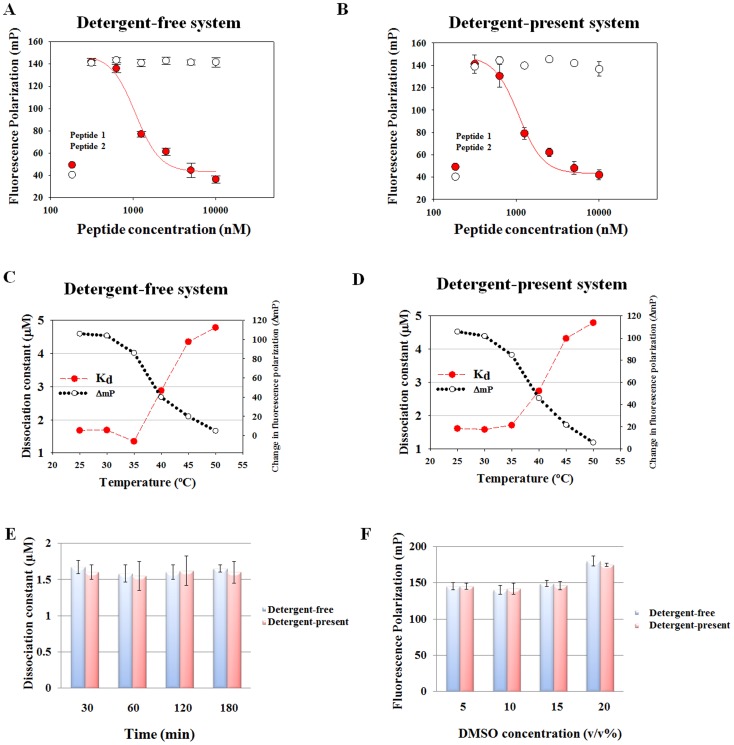
Assay development and stability assessment. A. Unlabeled peptide 1 (Ac-PFKPP-NH2) and mutant peptide 2 (Ac-PAKPP-NH2) were titrated into a mixture of c-Cbl (RING) protein (3 µmol/L)+100 nmol/L probe 1 separately in the detergent-free system. IC_50_ (peptide 1) = 1.05±0.09 µmol/L. B. Unlabeled peptide 1 and mutant peptide 2 were titrated into a mixture of c-Cbl (RING) protein (3 µmol/L)+100 nmol/L probe 1 separately in the detergent-present system. IC_50_ (peptide 1) = 1.06±0.09 µmol/L. C and D, temperature dependence of K_d_ and ΔmP values in detergent-free or detergent-present system. c-Cbl (RING) protein was titrated against 100 nmol/L probe 1. E. Assessment of stability of binding experiments over time in detergent-free or detergent-present system. c-Cbl (RING) protein was titrated against 100 nmol/L probe 1. FP values were measured at 25°C up to 180 min. F. Stability of binding experiments with increasing DMSO concentrations in detergent-free or detergent-present system. DMSO was titrated into constant amounts of c-Cbl (RING) protein (3 µmol/L) and probe 1 (100 nmol/L) mixture. The total reaction volume was maintained constant in all wells at 20 µl. The measurements were made at room temperature (25°C) after 30 min of incubation.

To determine whether probe **1** binds to the active site of c-Cbl(RING) protein and to choose a positive control, competition assays were carried out using peptide **1** (Ac-PFKPP-NH_2_) or mutant peptide **2** (Ac-PAKPP-NH_2_) (**Fig. 2**). We predicted that peptide **1** will be able to replace probe **1** and peptide **2**, which lacks the active phenylalanine residue, will not be able to do so. The addition of peptide **1** was shown to cause the return of the FP values to the level of free probes (**Fig. 2A**) which indicates the ability of peptide **1** to replace probe **1**. The IC50 value for probe 1 binding to c-Cbl(RING) protein in detergent-free system is 1.05±0.09 µmol/L. However, the addition of mutant peptide **2** does not alter the FP values, which is consistent with the prediction (**Fig. 2A**). To determine whether adding detergent to the assay buffer would change the dynamic stability, assays conducted in Figure 2A have been repeated under same conditions except the buffer contains 0.01% Triton X-100. The IC_50_ value for probe **1** binding to c-Cbl(RING) protein in the presence of detergent is 1.06±0.09 µmol/L (**Fig. 2B**) which is comparable to the IC_50_ value in detergent-free system. Probe **2** does not bind to c-Cbl(RING) protein under detergent-present condition (**Fig. 2B**).

To optimize the FP assay properly, it was necessary to assess the stability of the assay. The conditions for the FP assay were established by testing various parameters including incubation temperature, time, TF values, ΔmP values, DMSO tolerance, and buffer components. The K_d_ values increased and ΔmP values decreased significantly when the plates were heated over 35°C, indicating the probe-protein system started to dissociate (**Fig. 2C & D**). Our data show that at room temperature (25°C) the systems are stable up to 3 h (**Fig. 2E**). Therefore, the incubation time was optimized to 30 min for rapid screening. The DMSO tolerance for the FP assay was also tested, and stable FP values (**Fig. 2F**) and TF values (data not shown) were observed at up to 15% DMSO in PBS buffer (v/v). In summary, the competitive FP binding assay conditions ideal for identifying c-Cbl (RING) domain regulator are: (1) 3 µmol/L c-Cbl (RING) protein, (2) 100 nmol/L probe **1**, (3) 5% final DMSO concentration (v/v), (4) incubation for 30 min at 25°C before reading the plate, (5) with or without 0.01% Triton X-100.

### Pilot Screens and Assay Performance Measurements

Pilot screens against the natural product library of 10,000 compounds described above was performed at screening concentrations of 10 µmol/L to 0.078 µmol/L (half serial dilutions) in 5% DMSO (v/v). The screens were completed in the detergent-free assay first and the detergent-present assay later. Controls present in each assay plate consisted of 5% DMSO (negative control) or peptide **1** (positive control). The assay was semi-automated and the steps are listed in **Table** **2**. The performance of the FP assays and post-screen analysis were summarized in **Table 3**. Assay performance was assessed using Z’ factor generated from whole DMSO plates.

**Table 2:** Screen assay protocol.

Step	Parameter	Value	Description
1	Plate master mixture	19 µl	Master mixture of c-Cbl (RING) protein and probe **1**
2	Controls	1 µl	10 µmol/L to 78 nmol/L titration series of peptide **1**
3	Library compounds	1 µl	10 µmol/L to 78 nmol/L titration series
4	Incubate time	30 min	Room temperature
5	Assay readout	Ex/Em = 485/538 nm	ZS-2 plate reader

Plates lidded until read.

**Table 3:** FP assay performance and post-screen analysis summary.

Category	Parameters	Descriptions
	Nature of the assay	Cell-free multicomponent competitive assay
Assay	Assay strategy	Detection of c-Cbl (RING) domain regulator using fluorescence polarization assay
	Reagents and sources	See materials and Methods
	Assay protocol	Key steps outlined in Table **2**
	Nature of the library	Fractions derived from natural Chinese medicine
	Size of the library	10,000 natural compounds arrayed in 96-well plates as single compounds at 10 mM in DMSO
Libraryscreened	Source	Pharmanic, China
	Quality control	All compounds assured by the lab as >98% pure on HPLC with QC data
	Concentration tested	5% DMSO for the initial screen; 8 concentration tested from 10 µmol/L to 78.125 nmol/L (half dilutions)
	Format	384-well plate
	Plate controls	Positive control: peptide **1**; negative control: 5% DMSO
	Plate number and duration	430 plates over 60 days
Screenprocess	Output, detector, analysis software	ZS-2 detector; Fixed endpoint; FP value, SigmaPlot
	Normalization	% inhibition = 100 × (sample result – average of negative control)/(average of negative control – average of positive control)
	Performance	Z’ = 0.62 (detergent-free system)Z’ = 0.73 (detergent-present system)
	Selection of actives	Actives were selected from the primary screen using a threshold of better than 5 µmol/L (IC_50_)
Post-screenanalysis	Retesting of initial actives	Original samples retested using screening assay condition; compounds with triplicated activity tested in dose-response mode (8 half dilutions)
	List of validated compounds	Table **4**

### Post Screen Analysis

We screened a library of 10,000 natural compounds for c-Cbl (RING) domain regulators. 3855 compounds were excluded from the post-screen validation in both detergent-free and detergent-present systems if the compounds exhibited: (1) auto fluorescence, (2) quenching of fluorescence, (3) precipitation from assay, or (4) a decrease in polarization signal while lacking a dose response. There are 422 compounds showed inhibitory activity at the concentration of 10 µmol/L. Five candidates, methylprotodioscin, leonuride, catalpol, tehmaglutin and quercetin, could generate a dose-response curve and have IC_50_ values less than 5 µmol/L (**Table 4**) were identified as hit compounds in the detergent-free system. There are only 12 compounds showed inhibitory activity at the concentration of 10 µmol/L. Three candidates, methylprotodioscin, leonuride, and Catalpol, were identified as hit compounds in the detergent-present system. All compounds have been tested in more extensive completive binding assays. Two of the compounds from the detergent-free screen, tehmaglutin and quercetin, were verified as false positive hits possibly due to the aggregation (**Table 4**).

**Table 4:** Summary of hit compounds.

Number	Compound	Derived from	IC_50_ (µmol/L)			
			Primary screen		Validated	
			DF	DP	DF	DP
**1**	Methylprotodioscin	Cochinchinese Asparagus Root	4.8	4.0	4.2±0.5	4.0±0.8
**2**	Leonuride	Rehmannia glutinosa	3.5	3.8	4.6±0.8	4.5±1.1
**3**	Catalpol	Rehmannia glutinosa	2.9	2.3	2.7±0.6	3.2±0.9
**4**	Tehmaglutin	Rehmannia glutinosa	5.0	>10	4.7±0.4	NR
**5**	Quercetin	Cuscuta chinensis Lam	4.2	>10	4.0±0.6	NR

DF: detergent-free system.

DP: detergent-present system.

NR: no dose response.

## Discussion

Design and development of c-Cbl regulators is an active field of study because c-Cbl is associated with multiple diseases including obesity, diabetes and cancer [8], [11]–[13]. There are reports of assays being developed to screen the c-Cbl TKB domain indictors [14], [15]. Studies show that the majority of the binding energy of protein-protein interactions is contributed by a few amino acids at the interface [16]. Therefore, molecules derived from natural or bioactive peptides produced by plants, animals or humans are good candidates for screening. The potency of these peptides can be increased by enhancing hydrophobic, electrostatic and hydrogen-bonding interactions between the peptide and protein target. Crystallization studies show that c-Cbl binds to UbcH7 with its RING domain and its linker helix [17]. The RING domain provides a shallow groove which interacts with the L1 and L2 loops of UbcH7. This c-Cbl groove provides contacts for UbcH7 which include Phe-63 of the UbcH7 L1 loop as well as Pro-97 and Ala-98 of the UbcH7 L2 loop [17]. The Phe-63 (blue) makes multiple van der Waals contacts with Ile-383 on the c-Cbl RING domain. It also makes contacts with Trp-408, Cys-404, Ser-407, and Ser-411 from the α helix of the c-Cbl RING domain (**Fig. 1A**). The Pro-97 colored in blue of UbcH7 L2 loop makes contact with Ile-383, Trp-408, and Pro-417 of c-Cbl. The carbonyl group of Ala-98 of UbcH7 L2 loop makes a hydrogen bond with amide group on Pro-417 of c-Cbl which is another potential binding interface for chemical regulators (**Fig. 1B**). Based on these studies, fluorescein isothiocyanate (FITC) was used to label peptides derived from UbcH7 L1 loop or L2 loop structure which mimic the binding sequence (**Table 1**).

FP is a solution-based, homogeneous technique requiring no immobilization or separation of reaction components and therefore is suitable for HTS assays. The principle of the screen is to identify the compounds that displace probe **1** from the c-Cbl (RING) protein by detecting the resulting decrease in FP. The fluorescent labeled peptide (probe **1**) bound to c-Cbl (RING) domain with a sub-micromolar dissociation constant was inhibited by peptide **1** (**Fig. 2A &2B**). Therefore, probe **1** can be used as the fluorescent labeled probe for the FP assay.

It is widely accepted that aggregation, autofluorescence, and reactivity of artifacts in a HTS for inhibitors are potential causes for false positives [18]–[20]. It is also recognized that a substantial fraction of small molecules or natural compounds exhibit aggregating behavior leading to false positive results in screening assays [18]. This nonspecific attachment to target protein could be dramatically reduced by adding small amount of detergent to the assay buffer [19]. Therefore, we compared two screen conditions: detergent-free and detergent-present (0.01% Triton X-100) to assess the best conditions for HTS. The assay stabilities including temperature, time, and DMSO tolerance were tested and compared. Both detergent-free and detergent-present systems are stable under the indicated conditions (**Fig. 2C, 2D, 2E & 2F**).

To determine which condition is suitable for HTS, two pilot screens have been done and compared side by side. There are 422 compounds showed activity at the concentration of 10 µmol/L in the detergent-free screen which is an unexpected high hit ratio. One of the hit compounds identified from the detergent-free screen, quercetin, has been reported multiple times as a false positive hit [19], [20]. There are only 3 compounds showed activities in the detergent-present system. Tehnaglutin and quercetin, two hit compounds identified from the detergent-free screen, turned out negative in the detergent-present screen which indicates the detergent-present condition is more efficient for identifying true hit compounds. It is very interesting that methylprotodioscin, catalpol, and quercetin have been reported to be effective for diabetes or cancer treatments [21]–[25]. These natural products have been long used in traditional Chinese medicine. However, the mechanisms of these drug actions are still largely unknown. Our data shed some lights on the possible mechanism of these compounds. These compounds could be studied in future experiments in order to identify their specific mechanisms.

In all, we report on the development of a fluorescence polarization based high-throughput assay to identify potential c-Cbl (RING) domain regulators. Based on the pilot screens and validation assays, we have confirmed the binding activities of the three hit compounds. Our data have proven this screen can be used as a fast, robust, and quantitative measure of binding affinity for a wide range of c-Cbl (RING) domain regulators. The quantitative nature of the data significantly aids the efforts to choose the hit compounds. The Z’ factor of 0.73 indicates this assay could be easily adapted for HTS. We predict that this assay will be suitable for identifying small molecule regulators of the c-Cbl (RING) domain.

## Supporting Information

Figure S1 **Schematic representation of domain structures of Cbl proteins.** Numbers indicate amino acid position.
(TIF)

Figure S2 **Schematic representation of c-Cbl WT and c-Cbl (RING) constructs used in this study.** Numbers indicate amino acid position.
(TIF)
