# Supplementary figures and images for: Development of a Fluorescence Polarization Based High-Throughput Assay to Identify Casitas B-Lineage Lymphoma RING Domain Regulators
Source: PLoS One. 2013 Oct 31;8(10):e78042. doi: 10.1371/journal.pone.0078042 (PMC3814989; doi:10.1371/journal.pone.0078042)

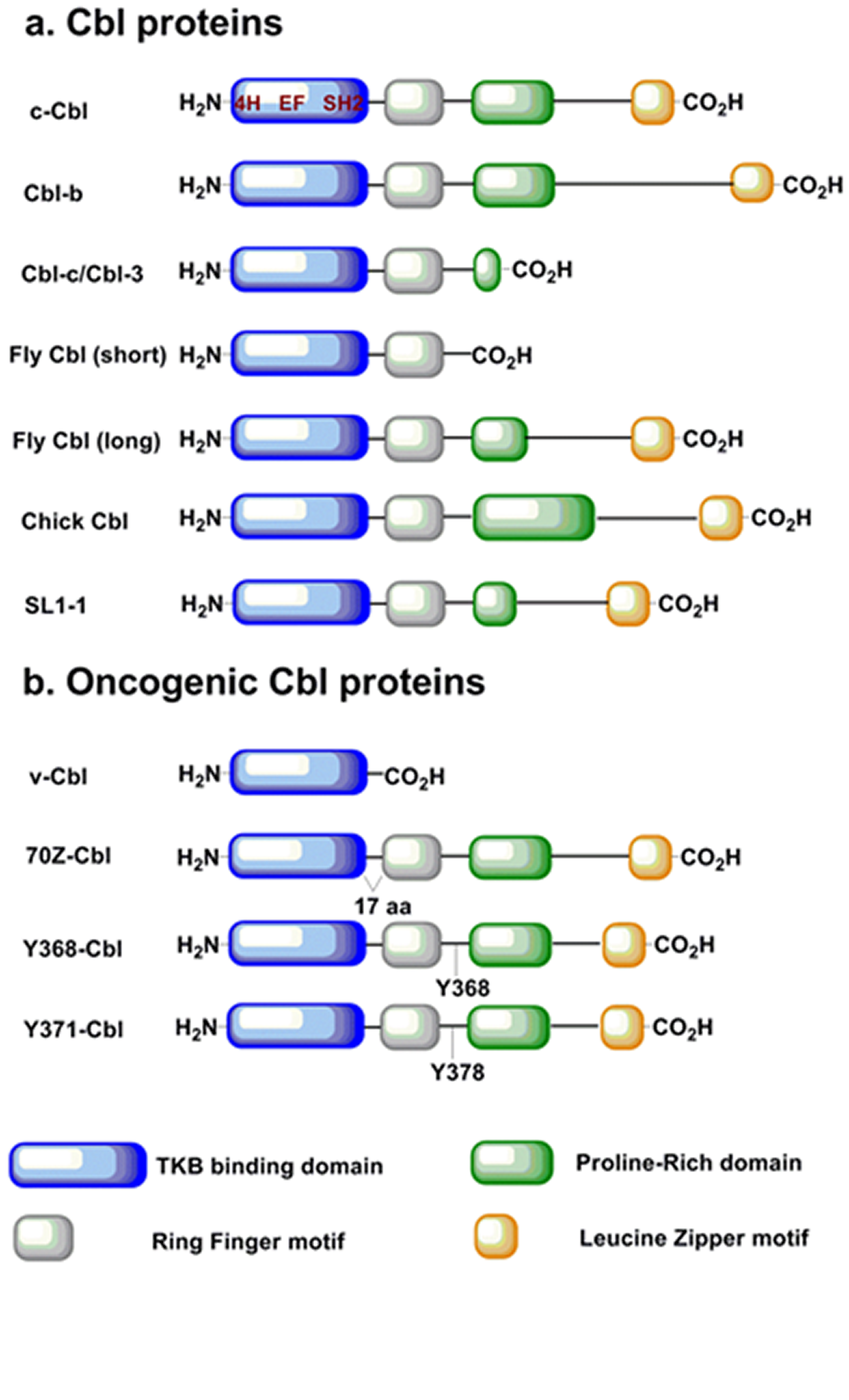

Supplement: Figure S1 — Schematic representation of domain structures of Cbl proteins. Numbers indicate amino acid position. (TIF) [file pone.0078042.s001.tif]
